# Resistance to complement activation, cell membrane hypersialylation and relapses in chronic lymphocytic leukemia patients treated with rituximab and chemotherapy

**DOI:** 10.18632/oncotarget.25657

**Published:** 2018-08-03

**Authors:** Anne Bordron, Cristina Bagacean, Audrey Mohr, Adrian Tempescul, Boutahar Bendaoud, Stéphanie Deshayes, Florence Dalbies, Caroline Buors, Hussam Saad, Christian Berthou, Jacques-Olivier Pers, Yves Renaudineau

**Affiliations:** ^1^ U1227 B Lymphocytes and Autoimmunity, Université de Brest, INSERM, IBSAM, Labex IGO, Networks IC-CGO and REpiCGO from ‘Canceropole Grand Ouest, Brest, France; ^2^ Department of Haematology, CHRU Brest, Hôpital Morvan, Brest, France; ^3^ Laboratory of Immunology and Immunotherapy, CHRU Brest, Hôpital Morvan, Brest, France; ^4^ Laboratory of Haematology, CHRU Brest, Hôpital Morvan, Brest, France

**Keywords:** chronic lymphocytic leukemia, rituximab, complement-dependent cytotoxicity, sialylation, progression-free survival

## Abstract

The anti-CD20-specific monoclonal antibody rituximab (RTX), in combination with chemotherapy, is commonly used for primary treatment in chronic lymphocytic leukemia (CLL). However, relapses remain important and activation of the complement pathway is one of the mechanisms by which RTX generates the destruction of B cells directly by complement-dependent cytotoxicity (CDC), or indirectly by antibody-dependent cellular phagocytosis. In this study, the RTX capacity to induce CDC was established in 69 untreated CLL patients, this cohort including 34 patients tested before the initiation of RTX-chemotherapy. *In vitro* CDC-resistance to RTX predicts lower response rates to RTX-chemotherapy and shorter treatment free survival. Furthermore, the predictive value of CDC-resistance was independent from the clinical, cytogenetic and FcγR3A V158F polymorphism status. In contrast, CLL cell resistance to CDC predominates in IGHV unmutated patients and was related to an important α2-6 sialyl transferase activity, which in turn increases cell surface α2-6 hypersialylation. Suspected factors associated with resistance to CDC (CD20, CD55, CD59, factor H, GM1, and sphingomyelin) were not differentially expressed or recruited between the two CLL groups. Altogether, results provide evidence that testing RTX capacity to induce CDC *in vitro* represents an independent predictive factor of therapeutic effects of RTX, and that α2-6 hypersialylation in CLL cells controls RTX response through the control of the complement pathway. At a time when CLL therapy is moving towards chemo-free treatments, further experiments are required to determine whether performing an initial *in vitro* assay to appreciate CLL CDC resistance might be useful to select patients.

## INTRODUCTION

Rituximab (RTX), a monoclonal antibody (mAb) directed against the B cell molecule CD20, was the first mAb to receive approval to be used in non-Hodgkin's lymphomas (NHL), and later in chronic lymphocytic leukemia (CLL) [1]. Efficacy of RTX varies from patient to patient and a higher rate of relapse has been observed in CLL patients compared to NHL [2]. Thus, the combination of RTX with the purine analogue, fludarabine, and the DNA alkylating agent, cyclophosphamide, represents the gold standard as first line treatment, although alternative agents such as bendamustine or chlorambucil can be proposed in combination with RTX [3, 4]. The novel combinations with RTX have become more and more effective, but relapses still remain common events, which may be attributed, in part, to CLL cell resistance to RTX.

RTX mediates different mechanisms of B cell death such as: antibody-dependent cellular cytotoxicity (ADCC); complement-dependent cytotoxicity (CDC); Ab dependent cellular phagocytosis (ADCP) through the clearance of C3b(i) opsonized cells by macrophages; and direct cell death [5–11]. ADCC activity is linked to the efficiency of Natural Killer (NK) cells to kill tumor cells, and it is notably dependent on the Fc gamma receptor (FcγR)3 polymorphism V158F [12–14]. Although ADCC is suspected to be the main mechanism of action with regards to RTX therapeutic efficacy and clinical outcomes [15–17], several authors suggested that activation of the complement pathway is also involved in the clinical response to RTX [18–21]. To further support the importance of the complement pathway, it was established that RTX therapeutic activity was strongly enhanced in CLL patients resistant to RTX after concurrent administration of fresh frozen plasma [22]. Last but not least, complement deficiencies are reported in CLL patients and suspected of limiting RTX efficacy *in vivo* [23].

Mechanisms controlling the RTX capacity to induce complement activation are multiple, incompletely understood and the results reported are conflicting. On one hand, the level of the target molecule CD20 appeared to be important, as well as the presence of complement regulatory molecules, which seem critical, based on the observations that blocking CD55 and/or CD59 functions are effective in increasing RTX-induced CDC [24, 25]. On the other hand, complement C1q molecule recruitment into lipid rafts mediated by RTX suggests that expression of ganglioside M1 (GM1), a marker of lipid rafts, can determine the susceptibility to RTX treatment as reported in NHL [26]. Another mechanism of resistance to RTX could be the sialylation of the cell surface. Sialic acid acts frequently *via* a α2-3 or α2-6 glycosidic linkage to galactose and N-acetylgalactosamine. Enzymes that support these sialylations belong to the sialyltransferases (ST) family, and they are unlikely to be expressed in normal B cells [27]. The presence of sialic acid is implicated in the recruitment of complement inhibitors [28], and, in turn, controls the mAb-capacity to induce complement activation [29].

In order to support our hypothesis that resistance to RTX-induced complement activation may have clinical implications, we decided to study *in vitro* B cell resistance mechanisms to CDC in 69 CLL patients including 34 who would benefit from RTX-chemotherapy. The predictive value of the *in vitro* CDC-resistance response was independent from the clinical, biological and cytogenetic characteristics of the patients. In contrast, when the CDC results obtained *in vitro* were compared to the clinical response, an association between CLL resistance to CDC and RTX-chemotherapy response was observed. We further highlighted the important role of CLL α2-6 ST activity and membrane sialylation in determining resistance and susceptibility to CDC.

## RESULTS

### CDC of CLL cells induced by RTX is delayed and inhibited by Eculizumab

**Figure 1:**
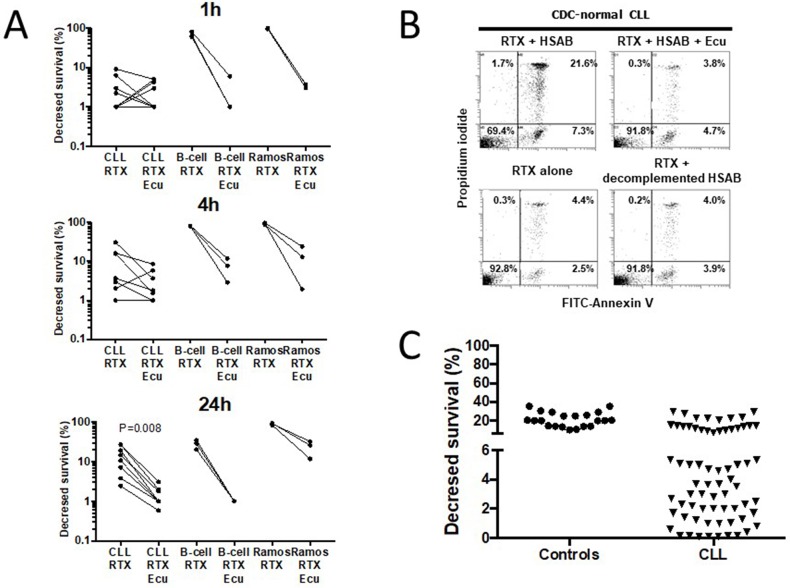
Rituximab (RTX)-induces *in vitro* complement-dependent cytotoxicity (CDC) of B lymphocytes in normal controls and in a subset of CLL **(A)** In order to improve the determination of CDC induction by RTX (10μg/ml) in CLL cells, CDC was initially established after 1h (top), 4h (middle), and 24h (bottom) incubation at 37°C in CLL cells (n=9), healthy control B cells (n=4), and the human B cell line Ramos (3 experiments) using normal human serum AB (HSAB, 20%) as a source of complement, and Eculizumab (Ecu, 10μg/ml) as a terminal complement inhibitor. **(B)** RTX capacity to induce CDC in a representative RTX-normal CLL patient was abrogated when adding Ecu, when complement present in HSAB was heat inactivated or when HSAB was omitted. **(C)** B cells from 21 normal controls and 69 CLL patients were incubated 24h with RTX (10μg/ml) and HSAB at 20%. After staining with FITC-annexin V plus propidium iodide, cells were analyzed by FACS and the CDC decreased survival calculated (see material and methods).

The RTX capacity to induce *in vitro* CDC has been addressed by several authors but with significant differences with regards to the source and number of B cells, the time and concentration of RTX, the amount of sera, and the method for CDC detection (Table 1). Accordingly, the assay to test complement mediated killing of CLL cells sensitized by the anti-CD20 mAb RTX (10μg/ml) at 37°C was optimized in a preliminary step and, as shown in Figure 1A, CDC induced by RTX was effective after 1h when using the human B cell line Ramos (n=3) but such an effect was delayed to 24h when using CLL cells (n=8) and B cells from healthy controls (n=3). We further established that the RTX capacity to lyse CLL cells disappeared when using Eculizumab as a terminal complement inhibitor, when complement present in HSAB (human serum AB) was heat-inactivated or when HSAB was omitted (Figure 1A/B). Next, in order to differentiate CDC-normal from CDC-resistant CLL cells, the 24h time point was selected and a cut-off value of 6% was established by considering the mean of decrease in CLL survival (21.2±7.6%) minus 2 standard deviations of normal B cells from 21 healthy controls (Figure 1C). Therefore, 45 out of 69 (65.2%) CLL patients were considered resistant to complement *in vitro* and this is independent from the RTX capacity to induce direct apoptosis and ADCC (Table 2).

**Table 1:** Overview of the different assays reporting complement dependent cytotoxicity assay with rituximab (RTX) in patients with chronic lymphocytic leukemia (CLL), non Hodgkin's lymphoma (NHL), and human B cell lines

Cell type (nb)	Number of cells/mL	RTX in μg/mL	Sera (%)	Total incubation time	Detection method	Lysis (%)	Comment	References
PBMC from CLL (n=33)	5×10^4^	10 μg/ml	25%	16h	Alamar	<10% in 58% 10-25% in 21% >25% in 30%	PBMC not purified B cells (CD20: 65-98%)	[65]
NHL and myeloma cell lines (n=3)	10^6^	10 μg/ml	25%	12h	Trypan blue and PI	100%		[75]
CLL (n=5)	2.5×10^6^	20 μg/ml	50%	2.5h	PI	Near 0%		[23]
CLL (n=4) and Daudi cell line (n=3)	5×10^6^	10 μg/ml	50%	0.25h	PI	CLL: 10% Daudi: 50%		[76]
CLL (n=11)	5×10^5^	100 μg/ml	6.25%	2h	LDH	Near 0%		[48]
EHEB (CLL, n=3) and Raji (n=3) B cell lines	5×10^4^	10 μg/ml	16.7%	6h	WST-1	EHEB: 0% Raji: <20%		[77]
CLL (n=16) Daudi (n=5) SU-DHL4 (n=5) Raji (n=5)	Not reported	10 μg/ml	20%	1h to 6h	C^51^ (CLL) & IP (cell lines)	CLL: 15% Daudi & SU-DHL4: 50-100% Raji: 0%		[78]
PBMC from CLL (n=6) and NHL (n=6)	10^6^	10 μg/ml	50%	30 min	IP	PBMC-CLL: 10% PBMC-NHL: 60%	PBMC not purified B cells	[79]
PBMC-CLL (n=11) Raji (n=4) cell line	10^4^	10 μg/ml	10%	90 min	C^51^	PBMC-CLL: 10 ± 8% Raji: 41 ± 4%	PBMC not purified B cells	[80]
CLL (n=7)	Not reported	10 μg/ml	50%	30 min	TOPRO-3	Near 0%, except one (78%)	1/7 responder	[81]
CLL (n=10) and Daudi (n=2)	10^7^-10^8^	100μg/ml	50%	15 min to 1 hour	TOPRO-3 and IP	CLL: 10% Daudi: 90%	CLL cells isolated using Ficoll-Paque	[82]

**Abbreviations:** PI: propidium iodide; nb: number of experiments; LDH: lactate dehydrogenase release assay; C^51^: Chromium-51 release assay; CLL: purified peripheral blood B cells from CLL patients; PBMC: polymorphonuclear B cells from CLL patients.

**Table 2:** Complement dependent cytoxicity (CDC), direct apoptosis, antibody dependent cell cytotoxicity (ADCC) and associated factors characteristic for CDC-normal and CDC-resistant chronic lymphocytic leukemia (CLL) patients

	Control B cells	CDC-normal CLL	CDC-resistant CLL	Normal *vs* Resistant
Decreased survival (%, CDC)	21.2±1.7 (21)^*^	16.1±1.4 (24)	2.0±0.2 (45)	*p*<10^-3^
Decreased survival (%, apoptosis)	4.4±2. 8 (8)	5.9±1.6 (9)	4.3±1.3 (8)	NS
Decreased survival (%, ADCC)		13.6±1.6 (6)	16.1±5.6 (6)	NS
CD20 (ABC units)	317,000±56,800 (6)	95,700±12,000 (9)	75,000±5,800 (39)	NS
GM1 (MFI)	2.5±0.25 (6)	2.6±0.2 (20)	2.3±0.3 (26)	NS
Sphingomyelin (MFI)	33.4±3.2 (6)	27.5±7.8 (8)	31.1±5.0 (16)	NS
CD55 (MFI)	11.6±0.9 (6)	8.7±1.6 (6)	10.7±2.1 (6)	NS
CD59 (MFI)	5.5±0.7 (6)	5.6±0.8 (6)	6.8±1.0 (6)	NS
Factor H binding (%)	20.4±4.8% (6)	13.3±3.0% (6)	18.3±4.2% (10)	NS
Sambucus nigra (MFI)	52±3 (6)	97±32 (6)	243±35 (8)	*p*=0.01
Maakia amurensis (MFI)	44.8±8.5 (6)	38.6±17.8 (7)	43.4±5.1 (28)	NS
α2-6 ST activity (OD, 9h)	0.721±0.011 (4)	0.730±0.009 (5)	0.981±0.017 (5)	*p*<10^-4^

**Abbreviations:** ND: not determined; ABC: antibody-binding capacities; MFI: mean fluorescence intensity; OD: optical density at 492nm, NS: not significant.

^*^The number of samples tested is indicated in brackets.

### Time to relapse (TTR) is reduced in the *in vitro* CDC-resistant CLL patient subgroup

When the whole population was taken into consideration (Table 3), no difference was observed between the CDC-resistant and -normal groups with regards to demographic, clinical and biological characteristics of the patients (age, sex, Binet stage, progression free survival [PFS], treatment free survival [TFS], lymphocytosis, lymphocyte doubling time [LDT], cytogenetic characteristics, and CD38 expression).

**Table 3:** Characteristics of the patients according to rituximab capacity to induce complement-dependent cytotoxicity (CDC)

	CDC-normal (n=24 including 11 treated)	CDC-resistant (n=45 including 23 treated)	Statistics
**Demographic factors**			
Age, mean±SEM	73±2	69±2	NS
Sex Male:Female	10:14	27:18	NS
**Clinical data**			
Binet A/B/C	7/13/4	13/23/9	NS
PFS, mean (months)^*^	53	72	NS
TFS, mean (months)^*^	96	108	NS
**Tumoral cells**			
Lymphocytosis	80±12	69±8	NS
LDT, mean (months)^*^	10	23	NS
CD38 >30%, n (%)	6/22 (27.3%)	10/43 (23.3%)	NS
**Cytogenetics, n (%)**			NS
Low risk	9/21 (42.9%)	18/39 (46.2%)	
Intermediate risk	5/21 (23.8%)	6/39 (15.4%)	
High risk	7/21 (33.3%)	15/39 (38.5%)	
**Therapeutic response**			
Complete response, n (%)	11/11 (100%)	15/23 (65.2%)	*p*=0.03
Partial response, n (%)	0/11	8/23 (34.8%)	
TTR, mean (months)^*^	>80	45	*p*=0.03
OS, mean (months)^*^	>80	87	NS
**Cytogenetics, n (%)**			
Low risk	5/11 (45%)	5/20 (25%)	NS
Intermediate risk	2/11 (18%)	4/20 (20%)	
High risk	4/11 (37%)	11/20 (55%)	
**Complement fractions at 1^st^ infusion**			
C3c low level	2/10	4/19	NS
C4 low level	2/10	0/19	NS
**IGHV UM/M**	0/5	8/6	*p*=0.04
**FcγR3A V158F, n (%)**			
FF	5/10 (50%)	11/20 (55%)	NS
VF	4/10 (40%)	8/20 (40%)	
VV	1/10 (10%)	1/20 (5%)	
**Treatment**			
RFC:RB	8:3	20:3	NS

**Abbreviations:** PFS: Progression free survival; TFS: Treatment free survival; LDT: Lymphocyte doubling time; n: number of patients; NS: not significant; RFC: rituximab fludarabine and cyclophosphamide; RB: rituximab bendamustine; TTR: time to relapse; OS: overall survival. ^*^Kaplan–Meyer survival analysis.

**Figure 2:**
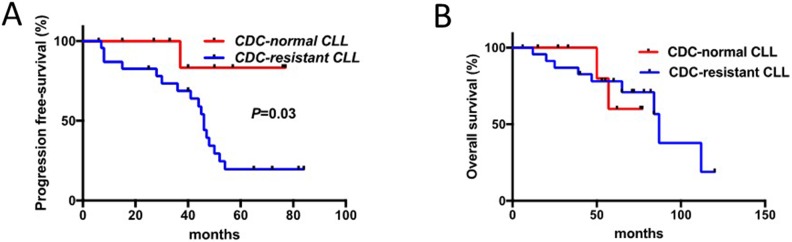
Response to therapy in CLL patients is associated with sensitivity to RTX-induced complement-dependent cytotoxicity (CDC) **(A)** TTR (time to relapse) of patients with B cells sensitive to RTX-induced CDC was compared to those resistant. **(B)** Overall survival (OS) of these two groups of patients was established and compared.

Further on, in patients tested before the initiation of RTX-chemotherapy for CDC (n=34), response to therapy was evaluated revealing in the CDC-resistant group a reduction when considering complete response (CR) (65.2% in the CDC-resistant patient group *versus* 100% in the CDC-normal patients group, *p*=0.03), and TTR (median TTR: 45 months in the CDC-resistant patient group *versus* >80 months in the CDC-normal patient group; Hazard Ratio 7.2, 95% CI 2.6-19.8, *p*=0.03) (Figure 2 and Table 3). The Cox regression model was used to determine the optimal cutoff for the CDC assay that was 6.7% and close to 6% as previously defined. In contrast, overall survival (OS) difference between the two groups was not significant due to the development of a secondary myelodysplastic syndrome leading to death in two patients from the CDC-normal patient group. Interestingly, our results further support an over-representation of CLL with unmutated IGHV in the *in vitro* CDC-resistant group (*p*=0.04), which is in agreement with Chai-Adisaksopha C and Brown JR report [30].

### Rituximab incapacity to induce CDC is not related to complement inhibitor levels, CD20 expression, FcγR3A V158F polymorphism or lipid membrane disorganization

**Figure 3:**
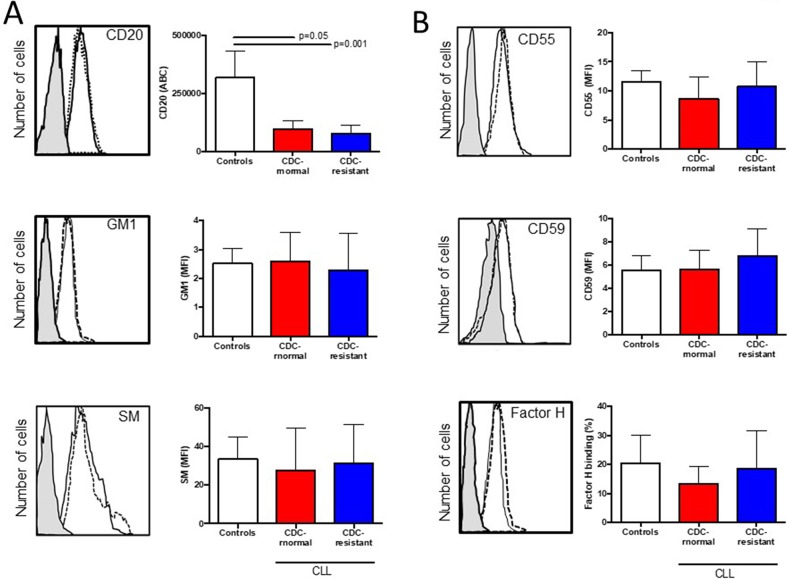
Expression of CD20, ganglioside M1, sphingomyelin (SM), and complement inhibitors (CD55, CD59 and factor H) in healthy control B cells and CLL cells according to their complement-dependent cytoxicity (CDC) status to rituximab (RTX) 5×10^5^ B lymphocytes were incubated with **(A)** anti-CD20 antibody, cholera toxin B identifying GM1 and lysenin identifying sphingomyelin (SM). **(B)** Same experiments were conducted with saturated concentrations of anti-CD55, anti-CD59 and anti-factor H antibodies. For each staining, a representative example is shown on the left side. The shaded histogram corresponds to isotype control, the solid line to a representative B cell staining from the CDC-normal patient group, and the dotted line to a representative B cell staining from the CDC-resistant patient group. The number of samples tested is indicated in Table 2 and differences are indicated when *p*<0.05.

Next we compared CD20 (target antigen), GM1 and sphingomyelin (lipid raft markers) *in vitro* expression between the two patient groups as the ability of CD20 to translocate into lipid rafts is suspected to be important for complement activation by anti-CD20 mAb [31], and that lipid raft integrity may be compromised in CLL cells [25, 26, 32] (Figure 3A and Table 2). No differences were observed when comparing in both groups (i) the numbers of CD20 molecules between groups; (ii) the mean fluorescence intensity (MFI) of the FITC-conjugated cholera toxin subunit B recognizing GM1; and (iii) the MFI of lysenin-bound-sphingomyelin. As we have previously observed that sphingomyelin overexpression in CLL cells induced by rifampicin affects the type II anti-CD20 mAb (B1) capacity to kill CLL cells [32], the experiment was repeated with RTX instead of B1 (tositumomab), revealing that sphingomyelin overexpression had no effect on the RTX capacity to induce CDC (data not shown).

Regarding complement regulators, we have further explored CLL cell surface expression of the complement inhibitors CD55 and CD59 [33], and CLL capacity to recruit the fluid-phase factor H, another RTX-mediated CDC inhibitor [34] (Figure 3B and Table 2). The MFI of CD55, CD59 and the percentage for factor H binding were assessed revealing similar values between the *in vitro* CDC-normal patient group and the CDC-resistant patient group. In order to complete such analysis, the complement deficiency at RTX-chemotherapy initiation was based on observing C3c and C4, as well as the cytogenetic status and FcγR3A V158F polymorphism known to be critical for proper CDC and ADCC, respectively (Table 3). No difference was reported and all these factors were not considered further.

### CLL lymphocytes CDC-resistant to rituximab express higher levels of terminal α2-6-linked sialic acids and their removal by neuraminidase increases activity of rituximab-induced CDC

**Figure 4:**
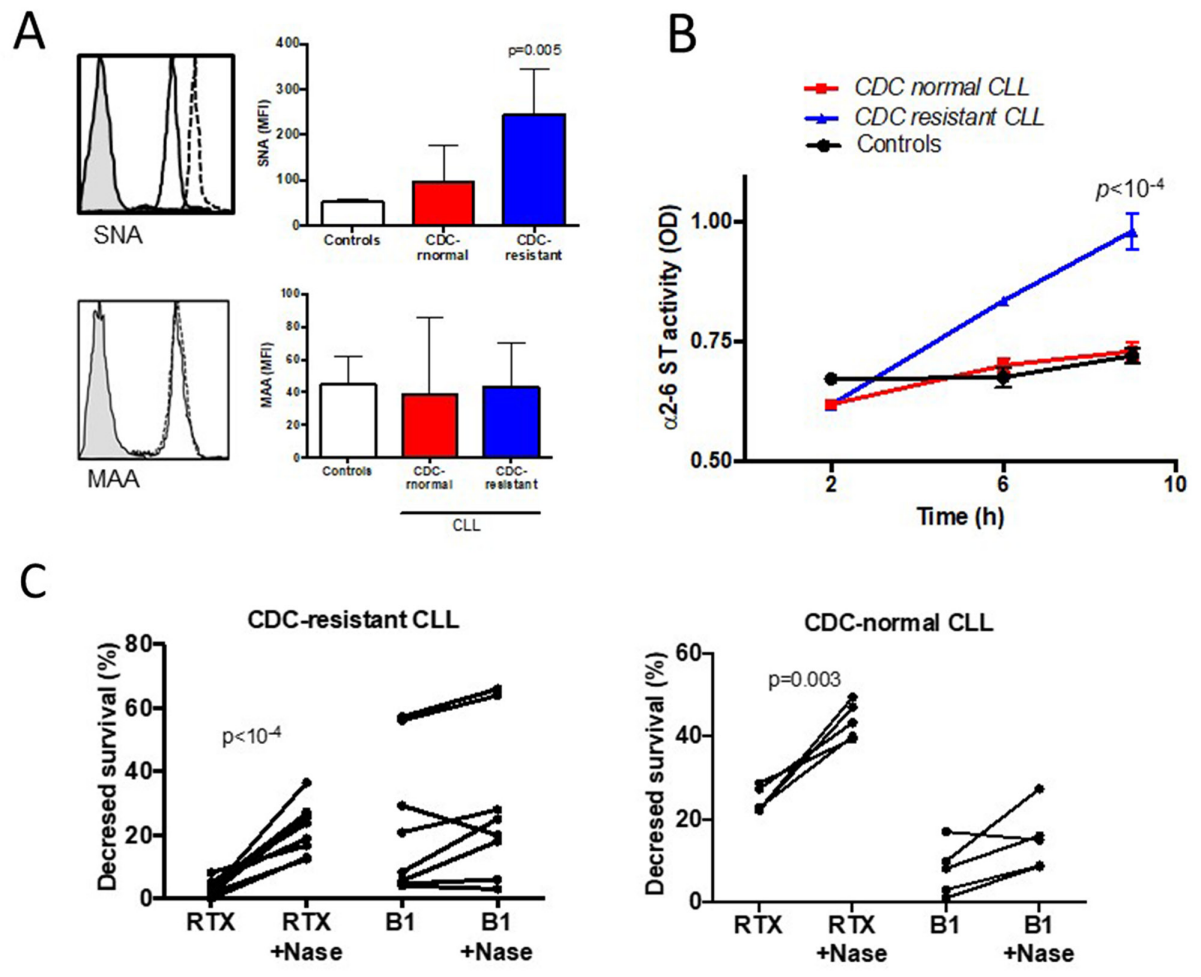
Hypersialylation characterizes CLL cells resistant to rituximab (RTX) induced complement-dependent cytotoxicity (CDC) **(A)** FACS analysis of α2-6 linked sialic acid stained with the lectin *Sambucus nigra* (SNA, upper panel) and α2-3 linked sialic acid stained with *Maakia amureusis* (MAA, lower panel). For each staining, a representative example is shown on the left side. The shaded histogram corresponds to isotype control, the solid line to CLL cells sensitive to RTX CDC, and the dotted line to CDC-resistant cells. **(B)** B cell lysates from five CDC-resistant patients, five CDC-normal patients and to the four controls were incubated on ELISA plates coated with an acceptor of sialic acid for 2, 6 or 9 hours. After washes, the biotinylated lectin SNA specific for sialic acid linked to α2-6 sialic acid was added. The results were visualized after incubation of streptavidin-conjugated horseradish peroxydase and 1,2 ortho-phenylenediamine. **(C)** B lymphocytes from ten patients within the CDC-resistant group and five patients within the CDC-normal group were treated with or without 0.05U of neuraminidase from *Clostridium perfringens*, washed and incubated with or without 10μg/ml of RTX or 10μg/ml of B1 (tositumomab) for 24 hours at 37°C. CDC was quantified by FACS after staining with FITC-annexinV and propidium iodide. Differences are indicated when *p*<0.05.

Because sialic acid residues from the cell surface have been shown to influence Ab mediated CDC in human carcinoma cells [29], the presence of α2-3 and α2-6-linked sialic acid was tested on CLL cells and controls using specific lectins. For this purpose, two lectins from *Sambucus nigra* (SNA) and from *Maakia amurensis* (MAA) were selected, which bind to sialic acid attached to terminal galactose in α2-6 and in α2-3, respectively (Figure 4A). The CLL cell membranes of the CDC-resistant patient group expressed more α2-6 sialylated proteins (MFI of 243±35) than those of the CDC-normal patient group (MFI of 97±32, *p*=0.01), and controls (MFI of 52±3). No differential expression of the lectin MAA was seen in the three different groups, suggesting similar levels of α2-3 sialylated proteins.

To test the hypothesis that α2-6 hypersialylation in the CDC-resistant patient group resulted from increased activity of α2-6 sialyl transferase (ST), we used a previously developed custom-made ELISA in order to measure the α2-6 ST activity in CLL cells [35]. As shown in Figure 4B, the α2-6 ST activity was found to be significantly upregulated in the CDC-resistant CLL cells treated (0.981±0.017) compared to the CDC-normal CLL cells (0.730±0.009, *p*<10^-4^) and to the B cell controls (0.721±0.011, *p*<10^-4^). In the MAA binding assay, the α2-3 ST activity was similar in the three groups (data not shown).

Finally, and to strengthen the effect of sialic acid on RTX-mediated CDC, we further selected 10 CLL patients from the CDC-resistant group and 5 patients from the CDC-normal group and cells were treated with neuraminidase to remove sialic acids from cell surface. In the CDC-resistant group, following neuraminidase treatment, RTX capacity to induce CDC was restored (*p*<10^-4^) (Figure 4C). Interestingly, the neuraminidase capacity to increase CDC activity was not restricted to the CDC-resistant subgroup as the same observation was made in the CDC-normal patient group (*p*=0.003). As a control and to test RTX specificity, when using B1 (tositumomab) instead of RTX, B1 capacity to directly kill CLL cells was similar following or not neuraminidase treatment (Figure 4C). Furthermore, since one may argue that desialylation can lead to membrane modifications and possibly to better accessibility of RTX to CD20 [36], we looked at the binding of RTX on the cell surface after desialylation. MFI of FITC-conjugated RTX binding to CD20 was similar with or without treatment with neuraminidase (data not shown). Altogether, these results suggest that treating CLL cells with neuraminidase can restore *in vitro* CDC induces by RTX, and differences between the two groups may be related to quantitative and/or qualitative differences in SA.

## DISCUSSION

Both clinical and experimental studies have offered evidence that resistance to RTX, rather than resistance to chemotherapy, controls relapse and clinical outcome in CLL. Among the different possibilities, complement activation could be the predominant mechanism for killing tumoral CLL cells *in vivo* and this notion is supported by several considerations. First, at initial infusion, side effects may be attributed in part to complement activation. Second, rapid exhaustion of complement and C3b(i) deposition on CLL cells was observed after RTX treatment in CLL patients and even more with Ofatumumab, another type I anti-CD20 mAb [37, 38]. Third, the addition of fresh frozen plasma enhances the effect of RTX-chemotherapy [39]. Fourth, intravital complement activation following anti-CD20 mAb administration is not limited to CDC but also to opsonisation priming mAb target cells for phagocytosis in ADCP [9, 40].

As different methods were used to determine the CDC mediated RTX, we first validated the *in vitro* model to measure the capacity of RTX to induce CDC. Tumor cell lines sensitive to RTX, inhibitor of complement activity and different times of RTX incubation were used. Based on the *in vitro* capacity of RTX to induce the complement pathway, two groups of patients were established. The first group, presenting CR (100%) and a delayed median TTR over 80 months, was sensitive to CDC *in vitro*, while the second group, with a reduced CR rate (65.2%) and a median TTR at 45 months, was resistant to CDC *in vitro*. Of note, TTR in the CDC-resistant group was similar to the TTR reported in 409 treatment-naïve CLL patients receiving fludarabine and cyclophosphamide [41]. Moreover, the higher rate of CR at the end of treatment in the CDC-normal patient group (100% *versus* 65.2%) further supports within the CDC-resistant patient group an incomplete capacity of RTX to activate the complement pathway. The critical contribution of complement activation in RTX-chemotherapy response is further supported by the absence of an association between the RTX response and the FcγR3A V158F polymorphism status, as previously described in CLL [42]. Next, other authors have attributed the differences in response to RTX treatment in CLL to IGHV mutational status [30] and to cytogenetic modifications and in particular to deletions at 11q/ATM or 17p/TP53 loci [43, 44]. In our study and with only one patient with 17p/TP53 deletion and three with 11q/ATM deletions treated with RTX-chemotherapy, more patients have to be included to test the unfavorable impact of these deletions on CDC mechanisms with anti-CD20 mAbs. The last parameter concerns the contribution of constitutive complement deficit in RTX-chemotherapy response to enhance TTR which is again not supported in our study by the determination of C3 and C4 components in sera of all CLL patients just before treatment initiation.

After determining that a longer culture time (24h) was required to allow CDC determination in CLL cells and having confirmed the absence of CDC response when using the anti-C5 mAb Eculizumab acting downstream C3 activation or when the HSAB was omitted or decomplemented by heat, important complement resistance mechanisms were suspected in CLL cells. After observing that CDC was restored in CLL cells after treatment with neuraminidase, it was alluring to incriminate sialylation as involved mechanism. Sialic acid is used in cancers, such as breast and ovarian carcinoma, as a protection against complement activity [29], and removal of sialic acid from the cell surface by sialidase increases sensitivity to CDC [45–47]. Applied to CLL patients, we further established that the α2-6 sialylation level influences resistance to the RTX-induced CDC patient group, and predicts the *in vivo* clinical RTX-chemotherapy outcome. Although a high level of sialic acid could favor the recruitment of complement inhibitors and consequently the resistance of CLL cells to RTX-induced CDC [34], we were unable to associate the increased level of α2-6 sialylation with factor H binding. It was recently demonstrated that inhibiting factor H binding, through the use of a novel mAb targeting factor H, sensitizes CLL cells resistant to RTX in a subset of patients [48]. This can be explained in part by the fact that factor H binds α2-3 but not α2-6 sialic acid [49]. Sialic acid is also known to recruit other complement regulators through binding of factor I, properdin and clusterin, which needs to be further explored. Whether these molecules are involved in CLL remains unknown. Nevertheless, sialylated proteins can directly induce resistance to complement by inducing inactivation of C3b [50], and in turn reduce the clearance of RTX opsonized cells by fixed tissue macrophages [10, 11]. An elevated level of sialic acid is one of the most effective mechanisms used by the target cells to control the ADCP process by macrophages [51]. Accordingly, our results support an abnormal quantity of sialic acid available on CLL cells to control both CDC and ADCP mediated by RTX. Further studies will be required to clarify the respective role of CDC and ADCP in the therapeutic response to RTX and further provide an explanation between the low level of CDC observed *in vitro* and its association with a better response. Similarly, as the phenomenon of trogocytosis mediated in particular by macrophages is described in the context of therapeutic antibodies this will need to be evaluated further with the limitation that trogocytosis controls partial loss of CD20 by RTX, but not CLL cell death and complement activation [52].

Elevated levels of the main galactoside α2-6 ST (ST6Gal-I) implicated in α2-6 sialic acid overexpression at the cell surface have been associated with metastasis and therapeutic failure in leukemia [53], and colorectal cancer [54]. Therefore, ST6Gal-I knockdown regulates tumor growth and invasion [55], and reverses protection against Fas mediated apoptosis [56]. A long list of sialylated receptors (e.g. CD24, CD43, CD45, CD52, CD62L, CD75) and receptors involved in sialic acid recognition (e.g. CD22) are described in B cells [57] and most of them are deregulated in tumoral B cells [58]. Treating CLL cells with neuraminidase was shown to reveal α2-6 sialic acid masked epitopes from the cell surface CDw75 [59], and the multidrug resistance protein 1 (MDR1) [60]. MDR1 overexpression is associated with therapeutic resistance in CLL [61], and mutations in MDR1 increase the risk of CLL [62]. As a consequence, future studies are necessary to test the impact of chemotherapy associated with RTX on α2-6 ST activity and whether blocking α2-6 ST activity and/or α2-6 sialic acid would improve RTX-chemotherapy not only by improving complement activation but also by reducing drug resistance. Another important question is related to the contribution of sialic acid overexpression on therapeutic response when using new generation anti-CD20 mAb. It's important to note that four patients from the CDC-resistant group have received Ofatumumab at relapse and that such administration was associated with an important C4 complement exhaustion [38]. A part of the patients from our cohort (47%) was also tested *in vitro* with the type II anti-CD20 mAb B1 (tositumomab) that triggers cell death by altering lipid organization in a mechanism independent from CDC and CLL hypersialylation ([32] and Figure 4C). Accordingly, future research is necessary to appreciate whether determining hypersialylation might be useful to propose a new generation anti-CD20 mAb as an alternative to RTX.

Other mechanisms controlling complement activation in patients receiving RTX-chemotherapy have been explored but with conflicting results reported when considering the absence of correlation between the complement inhibitors CD46, CD55 and CD59 levels and the RTX clinical outcome in NHL and CLL [18, 63], while blocking the complement regulatory molecules CD55 and CD59 improves RTX efficacy. Regarding the expression of the complement inhibitors, our results performed at baseline failed to highlight significant differences in agreement with some reports [64], but not all [18, 65] and this could be explained in part by different methodological strategies, patient selection and the fact that we have not tested CD55 and CD59 expression in the resistant CLL cells that were not cleared from the blood at completion of therapy [18]. As a consequence, the possibility that other factors control CDC needs to be taken into consideration. For this purpose, we have considered the capacity of the tumor cells to express cell membrane complement regulators, to recruit the fluid-phase complement inhibitor factor H [66], the level of the targeted antigen CD20, and the appropriate distribution of lipid rafts necessary for the recruitment of the complement protein C1q [31]. Fixation of RTX to CD20, density of CD20 molecules, GM1 and SM levels, complement inhibitor proteins CD55, CD59 and Factor H were evaluated in CLL cells in the two groups of patients.

Although these preliminary data are promising, this study has potential limitations. The developed *in vitro* assay necessitates long term incubations with RTX and we could not exclude that chemotherapy protocols have an effect on sialylation and CDC regulator expression. The sample size is quite small and is heterogeneous with respect to treatments. The analysis of the relationship between CDC status and clinical response were not adjusted for treatment, cytogenetic status, and immunoglobulin mutational status. In future studies it will be important to investigate dependence to these factors because existing data suggest that they are contributing factors in CLL relapse.

In conclusion, our main findings are: (i) part of the lytic effects of RTX-chemotherapy on tumor CLL cells is related to the capacity of RTX to induce the complement pathway; (ii) CDC is influenced by the level of cell surface α2-6 SA; (iii) a stronger activity of α2-6 ST characterizes the CDC-resistant patient group; and (iv) resistant capacity to CDC is not associated with distinct individual genetic groups and clinical outcome. From the therapeutic point of view and as CLL therapy is moving towards chemo-free treatments, future experiments are necessary now to develop the most appropriate biomarker between the three assays performed during this study: the CDC assay, α2-6 ST activity, and α2-6 sialic acid expression, in order to establish whether CDC resistance evaluation would help to determine early on those patients not likely to benefit from the classical first line FCR therapy.

## MATERIALS AND METHODS

### Patients and normal controls

Sixty-nine untreated patients fulfilling CLL diagnostic criteria were enrolled in this retrospective study including 34 tested before the RTX chemotherapy initiation (0 to 7 months) [67]. Disease assessment included Binet stage determination, CD38 expression, lymphocyte counts, LDT, and cytogenetic analysis as previously described [68, 69]. PFS was defined as the time from disease discovery to disease progression. Disease progression was considered either as the shift from Binet stage A to Binet stage B/C or as a short LDT of less than 6 months. TFS was defined as the interval between the date of disease discovery and the date of treatment initiation. Patients were segregated into cytogenetic risk groups according to the Döhner's classification [70]: patients with isolated del(13q) were in the low risk group, normal karyotype or trisomy 12 were in the intermediate risk group, and del(11q), del(17p) or complex karyotype were in the high risk group. RTX was introduced as first line treatment in combination with fludarabine and cyclophosphamide (RFC, n=28) or with bendamustine (RB, n=6). Response to therapy was assessed according to the 2008 IW-CLL guidelines [71] by determining the CR at the end of RTX infusions, TTR was defined as the time from RTX initiation to the time of disease progression (≥5 ×10^9^ peripheral B cells/L), and the OS rate from the inclusion to death from any cause. Blood was also withdrawn from 21 healthy volunteers. Consent was obtained from all individuals and the protocol approved by the Ethical Board at the Brest University Medical School Hospital (https://clinicaltrials.gov/ct2/show/NCT03294980; CRB Brest, collection 2008-214), in accordance with the Declaration of Helsinki.

### Specimen collections and complement analysis

Blood samples were collected in order to test the RTX capacity to induce CDC and for cellular analysis. Plasma was stored at −80°C until tested for C3c (normal range (NR): 0.81-1.57 g/L) and C4 (NR: 0.13-0.39 g/L) by turbidimetry (Spa-Plus^®^, The binding site group limited, Birmingham, UK).

### Cell preparation

Peripheral blood mononuclear cells were separated by density-gradient centrifugation on Ficoll-Hypaque, and B lymphocytes were isolated using a B-cell isolation kit according to the manufacturer's instructions (Stem Cell Technologies, Grenoble, France). The purity estimated by flow cytometry using FITC-conjugated anti-CD19 and PE-conjugated anti-CD5 mAbs was over 95%.

### Complement-dependent cell cytotoxicity assay

Based on our preliminary step optimization (Figure 1), RTX-induced CDC was evaulated *in vitro* as follows. Briefly, 3×10^5^ B lymphocytes were incubated into 24-well plates at 37°C in RPMI-1640 (Sigma-Aldrich, Saint-Quentin Fallavier, France) supplemented with 2mM L-glutamine, antibiotics, 10% fetal calf decomplemented serum (Gibco, Paisley, Scotland), and 20% of HSAB decomplemented or not 20 min at 56°C (Invitrogen, Cergy Pontoise, France). In all protocols 10μg/ml RTX-stimulation (Roche, Paris, France) (saturating Ab conditions [72]) with or without 10μg/ml Eculizumab (Soliris; Alexion Pharmaceuticals, Cheshire, CT), B lymphocytes were collected, washed and stained for 15 minutes with FITC-conjugated annexin-V (AV) and propidium iodide (PI) using the Beckman-Coulter apoptosis kit. The percentage of CDC decreased survival was calculated by using the formula: 100x [(% of AV-PI- cells without RTX in HSAB)-(% of AV-PI- cells with RTX in HSAB)]/[% of AV-PI- cells without RTX in HSAB]. In selected experiments, direct apoptosis and ADCC [25, 32] were monitored as previously described; and cells were additionally treated with 0.05U of neuraminidase (Sigma-Aldrich) for 30 minutes at 37°C, and two washes were then performed to avoid any side effects of neuraminidase on RTX or B1 (10μg/ml, generous gift of Cragg MS, Southampton, UK) activity.

### Mutational status of *IGHV*

As previously described [68, 69], the *IGHV* gene mutation status was determined by sequencing after conducting a PCR multiplex amplification. Briefly, for multiplex PCR, 100ng of genomic DNA, 0.25μl of Ampli Taq Gold DNA Polymerase (Applied Biosystems, Foster City, CA, USA), 10pmol of each primer, 0.2mM dNTP Mix, 1.5mM MgCl2, 1x PCR Buffer II, were adjusted to 50μl with DNase/RNase free ultrapure distilled water. Next, PCR products were visualized on 2% agarose gel, and purified with ExoSAP-IT PCR product cleanup kit (Affymetrix, High Wycombe, UK). Finally, amplicons were sequenced with a Big Dye Terminator v3.1 cycle sequencing kit (Applied Biosystems). Results were analyzed with the database IMGT/HighV-Quest (The international ImMunoGeneTics information system, Montpellier) and a homology sequence >98% defined an UM status.

### Polymorphism analysis in FcγR3A V158F

The BioSprint 15 DNA blood kit was used to extract genomic DNA from mononuclear cells according to the manufacturer's instructions (Qiagen, Valencia, CA, USA). Polymorphisms were determined as previously described [38, 73] using allele-specific polymerase chain reactions (PCRs) with one unmodified primer for FcγR3a-158V/F (ATATTTACAGAATGGCACAGG) and one locked nucleic acid modified primer for FcγR3a-158V: GAAGACACATTTTTACTCCCAA+C *versus* for FcγR3a-158F CTCTGAAGACACA-TTTTTACTCCCAA+A.

### Flow cytometry

In this study, 5×10^5^ freshly isolated lymphocytes were incubated for 30 minutes at 4°C with saturating concentrations of anti-CD19 mAbs combined with phycoerythrin (PE)-cyanin 7 (PCY7), and another mAb (anti-CD5, anti-CD20, anti-CD59 or anti-CD55) conjugated with fluorescein isothiocyanate (FITC). All mAb, as well as their isotype control mAb were from Beckman Coulter (Villepinte, France), biotinylated lectin SNA, specific to the α2-6 linked SA, and biotinylated lectin MAA specific to the α2-3 linked sialic acid (Vector Burlingame, CA), lysenin recognizing SM (PeptaNova, Sandhausen, Germany) or FITC-conjugated cholera toxin subunit B recognizing GM1 (Sigma-Aldrich). Samples containing biotinylated-lectin were covered for an additional 30 minutes with FITC-conjugated streptavidin (Jackson Immunoresearch Europe Ltd, Suffolk, UK). Cells stained with lysenin were incubated for 30 minutes with a polyclonal rabbit anti-lysenin Ab, and for another 30 minutes with FITC-conjugated anti-rabbit mAb (PeptaNova, Sandhausen, Germany). To study the binding of factor H on CLL cells, 3.10^5^ cells were incubated for one hour at 37°C with normal human serum diluted 1/4 in PBS followed by a 30-minute incubation with FITC-conjugated anti-factor H mAb (Abcam, Paris, France) at 4°C. The cells were then washed with phosphate buffer saline (PBS) and analyzed on FC500 flow cytometer (Beckman Coulter). The calculation of the MFI of all markers required a minimum of 5,000 events.

The number of CD20 molecules per cell was quantified by determining the amount of mAb binding to the cells at saturating concentrations, using the Quantum kit (Flow Cytometry Standards Corp).

### Assay for sialyltransferase activity

The activity of α2,6 and α2,3 sialyltransferases was evaluated by ELISA after B cell lysis as previously described [35] and the optical density (OD) was determined by a spectrometer ELISA at 492 nm.

### Statistical analysis

Continuous data are described as mean ± standard error of the mean (SEM). Following normality and equality of variance tests, nominal values were compared using the student's *t* test or alternatively by using a nonparametric test (Mann–Whitney rank sum test). For categorical data, differences among groups were analyzed using the Fisher's exact test. The profile likelihood method using a Cox regression model of TTR was used in univariate analysis to determine the optimal threshold, this analysis was computed using the Survival and SurvMisc R packages [74]. LDT, TFS, PFS and TTR analyses were performed using Kaplan–Meier curves and prognosis differences between groups were assessed with a log-rank test. P values under 0.05 were considered significant. Statistical analyses were performed using GraphPad Prism 7.0 (La Jolla, CA, USA).

## Abbreviations

ABC: antibody binding capacities
ADCC: antibody-dependent cellular cytotoxicity
CD: cluster of differentiation
CDC: complement-dependent cytotoxicity
CLL: chronic lymphocytic leukemia
CR: complete response
CRB: center for biological resources
FcγR3: Fc gamma receptor 3
GM1: ganglioside M1
HSAB: normal human serum AB
MAA: *Maakia amurensis*
mAb: monoclonal antibody
MFI: mean fluorescence intensity
NHL: non-Hodgkin's lymphomas
NK: natural killer
NR: normal range
NS: not significant
OD: optical density
OS: overall survival
PFS: progression free survival
PR: partial response
RB: rituximab bendamustine
RFC: rituximab fludarabine cyclophosphamide
RTX: rituximab
SA: Sialic acid
SD: standard deviations
SNA: *Sambucus nigra*
ST: sialyltransferases
TFS: treatment free survival
TTR: time to relapse
